# The perceived potential of religion in mitigating climate change and how this is being realized in Germany and Switzerland

**DOI:** 10.1007/s13412-023-00884-z

**Published:** 2024-01-12

**Authors:** Adam X. Hearn, Fabian Huber, Jens Koehrsen, Ann-Lea Buzzi

**Affiliations:** 1 Center for Religion, Economy and Politics, University of Basel, Basel, Switzerland; 2 Faculty of Theology, University of Oslo, Oslo, Norway

**Keywords:** Religion, Sustainability, Climate change, Religious organizations

## Abstract

Scholars of religion have repeatedly debated and contested the role of religion and spirituality in combatting climate change. In recent years, the potential of religion has also become an issue among natural scientists, politicians, environmental organizations, and civil society. Indeed, the potential of religion to mitigate climate change is perceived both internally and externally, and various expectations are placed on religion. This article examines the perceived potential of religion in mitigating climate change and how this is being realized. Based on 38 interviews, conducted with representatives from religious communities and umbrella organizations in Germany and Switzerland, we focus on the areas of values, political influence, and materialization. Our results show that the potential of religion in addressing climate change remains largely unfulfilled despite increasing steps in this direction.

## Introduction

The potential of religions for mitigating the climate crisis has been examined from a variety of perspectives (Hitzhusen and Tucker 2013; Harmannij 2019, 2022; Christie et al. 2019; Preston and Baimel 2021). However, this has been problematized (Veldman 2019), as religions can motivate strong convictions both against and in support of pro-environmental action (Preston and Baimel 2021). In this paper, we describe the expectations that are placed on religions when it comes to environmental engagement with an emphasis on climate change mitigation. Based on these expectations, we study forms of religious climate engagement taking place in two European countries, Switzerland and Germany. This contributes to social science climate change research by providing a better understanding of the ways in which religious organizations may contribute to climate change mitigation. It further provides a contribution to religious studies by shedding light on some of the social expectations concerning religion and climate change.

There is debate on whether overall, religions are greening, with indications that *some* sections of *some* religions may be engaged in certain forms of climate change mitigation (Koehrsen et al. 2023). However, other sections may be disengaged and sceptical of the climate crisis (Carr et al. 2012; Ecklund et al. 2017; Veldman et al. 2021), and yet others attribute the climate crisis to God, to be either addressed through prayer (Taylor et al. 2016), or even welcomed, as is the case for some apocalyptic evangelicals (Veldman 2019; Bjork-James 2023; Zaleha and Szasz 2015). Christian religious groups may frame climate change (often implicitly) from broadly two approaches (Clingerman and O’Brien 2017). Firstly, as an entirely new kind of problem calling for innovative solutions but also conversely enabling apocalyptic world views as well as the creation of new religions. Secondly, as a problem which is prefigured by other major historical challenges such as World War II (Clingerman and O’Brien 2017). The latter approach emphasizes the continued relevance of religion in providing solutions and building upon prior commitments to social justice and was more prevalent in the religious communities we interviewed.

Research in religious environmentalism indicates that congregations may not always implement the greening strategies of their umbrella organizations (Torabi and Noori 2019). Therefore, a focus which examines both umbrella organizations and congregations provides fertile ground as a basis for study (Koehrsen et al. 2022; Koehrsen and Huber 2021). Accordingly, our research focus is on both congregations and religious umbrella organizations. Congregations are social institutions in which individuals gather on a regular basis for events and activities with explicitly religious content and purpose (Chaves 2004). As such, congregations are the centre of religious community activities. They usually involve local religious leaders (e.g., pastor and imam), congregational staff (paid employees or non-paid volunteers), and members, participating to different extents in the congregational activities. Religious umbrella organizations are head organizations for a certain religious faith in a certain geographical space. They cover congregations related to this faith in the given space.

Henceforth, for the purpose of this research, we use the term “religious communities” to encompass both congregations *and* umbrella organizations.

We structure our paper by providing an overview of research on the expectations placed on religions in the next section, followed by a short section on the distinct religious landscapes of Germany and Switzerland. We then detail our methods and provide relevant results which are then further analyzed in “Discussion,” before concluding in our outlook.

## The expectations conferred upon religions

Studies on religion and the environment received significant interest following White’s (1967) article “The Historical Roots of our Ecologic Crisis.” White argued that Western Christianity with its anthropocentricism and its “dogma of man’s transcendence of, and rightful mastery over, nature” (1206) contributed to the ecological crisis. This dogma facilitated the development of ever-more invasive technologies (e.g., agricultural technologies). Combined with growing technological possibilities that allowed humans to massively intervene in nature, Western Christianity is portrayed as one of the central causes of extensive environmental destruction. However, he asserted that religions must also be part of the solution to the crisis, concluding.

“*Since the roots of our problems are so largely religious, the remedy must also be essentially religious*” (White 1967: 1207).

In recent years, there has been an increase in research on religion and ecology with some focus on religion and climate change (Jenkins et al. 2018: 86). This has been coupled with increased reflection on the potential of religion in combating climate change (Gardner 2003, 2006; Posas 2007; Haluza‐DeLay 2014; Jenkins et al. 2018; Koehrsen 2018; Ives and Kidwell 2019). Based on the literature, it is possible to outline three areas in which the various potentials of religion are visible: A) materialization, B) shaping values, and C) political influence (as a moral authority and with corresponding connections).

A) Materialization

Religious actors often have vast financial resources (Grim and Tucker 2014), as well as significant material and human resources. This enhances the potential of religions to engage in the fight against climate change, given that more than 80% of the global population are part of a religious tradition (Pew Research Center 2017). However, it should be noted that ascribing people to a single religion is also a simplification and not without contention (Lawson and Ramey 2018). Religions are not necessarily exclusive of each other (i.e., it is possible to follow more than one religion simultaneously), and criteria used to determine belonging are often contested (Ramey 2007).

Religious actors can also work to mitigate climate change by initiating projects to reduce their own CO_2_ emissions. This may involve the introduction of renewable energies (e.g., the installation of solar panels) or more efficient use of resources (e.g., reduced heating output in buildings, purchasing and consuming regional, sustainable products, or recycling) (Gottlieb 2006; Koehrsen 2015, 2018). Although this is directly related to human and financial resources, reducing energy consumption involves drawing on these resources but can also result in an increase in future financial resources owing to the potential savings made through energy efficiency measures and renewable energy generation.

B) Shaping values

Many scholars emphasize that religions shape people’s values and can thereby help mitigate climate change, creating a more environmentally sustainable society (Grim and Tucker 2014; Koehrsen 2018). For Tucker and Grim (2001), religions are the central shapers of values. Some authors argue that value dissemination is religion’s specific contribution to the fight against climate change, as politics, science, and economy cannot take over the dissemination of values and worldviews (Gardner 2006; Tucker 2006; Posas 2007; Gottlieb 2008; Bergmann 2009), although this has been challenged (c.f. Baugh 2017; Gade 2023). By spreading pro-environmental values and worldviews through preaching and religious education, religious communities can influence the lifestyles of broad segments of the population (Shibley and Wiggins 1997; Djupe and Hunt 2009). Social networks available to religious organizations via their members increase the potential reach of any climate mitigating action (Gardner 2003, 2006; Haluza‐DeLay 2014). Furthermore, by offering compelling reasons to resist the spread of consumerism, religions could turn environmental actions into flags around which society can rally (McKibben 2001). These arguments have been contested (Baugh 2017; Gade 2023) indicating that religion may play also a negative role or no role at all in shaping environmentally friendly values. An example of this is research from Poland that indicates that urban, better-educated, less religious people are more open towards notions of an energy transition than those that live more rurally, are less educated and are more religious (attend church regularly) (Żuk et al. 2021).

C) Political influence, connections

The public dimension of religion enables further potential regarding political influence (cf. Casanova 1994). Religious communities may have a wide reach and can influence on public opinion, which allows them to take a stand on environmental issues (Gardner 2003; Tucker and Grim 2001). According to Hoffman (2015), the reach of major religious figures such as the Pope may be much greater than that of other public figures such as politicians. This is reinforced by the connections that religious actors have to policymakers (Haluza‐DeLay, 2014). Thus, religion can engage using public visibility and political influence to promote (or hinder) environmental protection through public statements, media exposure, and lobbying of decision makers (Gardner 2006; Wardekker et al. 2009; Johnston 2010; Glaab 2022; Huber 2022).

Research has been conducted on how religion/religiosity affect the environmental behavior of individuals, showing different effects of religious attitudes on environmental behaviour (Greeley 1993; Kanagy and Willits 1993; Woodrum and Hoban 1994; Kanagy and Nelsen 1995; Wolkomir et al. 1997; Woodrum and Wolkomir 1997; Biel and Nilsson 2005; Sherkat and Ellison 2007; Djupe and Gwiasda 2010; Barker and Bearce 2013; Gutsche 2019). Some have suggested a negative influence of religion on attitudes of adherents towards climate change. This may be due to a focus in research on religious conservatives which are often associated with climate change denial (Szasz 2012; Veldman 2019). At the same time, there is evidence to show that this is not a static state and that religious conservative beliefs regarding climate change shift throughout time (Veldman et al. 2021; Hempel and Smith 2020).

Other studies (Buckley 2022; Myers et al. 2017; Maibach et al. 2015) also attribute a positive effect to religion and thus point to their potential for instigating climate change mitigatory action, such as through the encyclical, *Laudato Si’* (Francis 2015). The encyclical, subtitled “*on care for our common home*,” examines topics such as consumerism and global warming and implores people to take remedial action. Studies on the encyclical *Laudato Si’* show that commitment increased following the encyclical (Buckley 2022), pointing to a positive effect on the behaviour of followers (and non-followers). These findings, also described as the “Francis Effect” (Maibach et al. 2015), suggest that *Laudato Si’* led many people with prior high levels of concern to take additional actions—that is, their attitude-behavior consistency increased (Arbuckle 2017; Myers et al. 2017; Schuldt et al. 2017). Nevertheless, this effect was limited to the politically liberal and did not take place among conservatives, who relativized the importance and authority of the Pope on climate change attitudes (Li et al. 2016; Landrum et al. 2017; Harmannij 2019; Berry 2022; Taylor et al. 2016; Danielsen et al. 2021). Ultimately, the assumption seems to be that religion does not play a decisive role. Positional references of religious actors seem to be interpreted on an individual level to fit into one’s own worldview. In other words, those that were already positively disposed towards environmentally friendly actions found added congruence whereas climate sceptics relativized the competence of religious actors in this area.

Overall, the results of research from the social sciences remain mixed. Even if often negative/no influence could be proven, studies on the encyclical in particular (Arbuckle 2017; Myers et al. 2017; Schuldt et al. 2017) show that religion has the potential to move people to act in more climate-friendly ways.

In the natural sciences, climate change is now recognized as a problem that cannot be solved with technology alone, and there is a growing understanding that cultural aspects need to be taken into account (Hulme 2017: 240–241). Accordingly, the latest IPCC report (IPCC 2022) also addresses and calls into action more diverse actors, including religious actors. The potential of religion is also explicitly mentioned: “Religion could play an important role in enabling collective action on climate mitigation by providing cultural interpretations of change and institutional responses that provide resources and infrastructure to sustain collective actions.” (IPCC, 2022: 570).

Influential climate scientists have also publicly called for enhanced collaboration among religious institutions, policymakers, and the scientific community, and the engagement of religious actors is often well-received (Dasgupta and Ramanathan 2014; Hayhoe, 2019). However, even if there are some opportunities and few major barriers to cooperation, religious actors are seldom directly addressed by the natural sciences (Hulme 2017; Harmannij 2019).

Internally, climate change influences religious discourse and is an important subject in theological debate (Jenkins et al. 2018), with corresponding theologies and statements from leaders and umbrella organizations^1^ (Harris 1995; Foltz 2006; Tucker 2008; Boff 2011; Saniotis 2012; Dessì, 2015; Blanc 2017; Chaplin 2016). Some scholars from the religion and ecology debate have interpreted these endeavours as leading to a “greening” of religions, suggesting that religions become more environmentally friendly over time (Tucker 2006, 2008; Chaplin 2016), although this is contested (c.f. Taylor et al. 2016).

The aforementioned *Laudato Si’* is a good example of religious discourse on climate change and is embedded in developments within Christianity. The encyclical was preceded by developments, such as the M Forum of the WCC in Nairobi (1975), the “*Justice, Peace and Creation Concerns*” commission in Vancouver (Gill 1983), and that Pope John Paul II and Pope Benedict as well as Patriarch Bartholomew were already active in this area (Tucker and Grim 2001; Blanc 2017, 2022).

Corresponding statements and actions on climate change are also evident in other religious traditions, for example, the Dalai Lama calls for action to mitigate climate change (Tucker and Grim 2001; Posas 2007; Haluza‐DeLay, 2014; Chaplin 2016; Hulme 2017; Jenkins et al. 2018), and both the Buddhism Faith Statement on Ecology (Fossey 2003), and the Hindu Declaration, issued calls to address climate change through personal transformation and public action.^2^ The Islamic Declaration on Climate Change puts forth the notion that all people are caretakers, calling for a “fresh model of wellbeing” (International Climate Change Symposium, 2015). The language used stops short of calling directly for climate justice, addressing multiple sectors of society regardless of religion in calling for a move to a sustainable circular economy and the scaling up of renewable energy amongst other things (Gade 2023). Yet, it may be questioned whether such public statements reflect the positions of broad sections of followers of the given religions or just the positions of a small elite of religious environmentalists (Gade 2019).

While these statements and actions may have been partially precipitated owing to external pressure regarding the potential of religion, they may increase social expectations on different religions to take up greater roles in mitigating climate change. In the cases of Switzerland and Germany, social expectations tend to be greatest when it comes to the largest established Christian religions, Roman Catholic, and Protestant.

## Religion in Switzerland and Germany 

Religious landscapes in Switzerland and Germany are characterized by secularization, pluralization, and individualization (Pickel 2011; Pollack and Olson 2012; Bochinger et al. 2012; Baumann and Stolz 2007). The two large Christian churches (Roman Catholic and Protestant) struggle with declining membership and the number of people who identify as “non-religious” is increasing. Growth is only found on a small scale, among certain evangelical communities with a charismatic background, among religious groups with a migrant background (e.g., Muslim and Buddhist communities), and among alternative spiritualities (Bochinger et al. 2012; Stolz et al. 2014, 2022; Becci 2022). The two main Christian churches represent the largest religious groups in both countries. In Germany, approx. 28% of the population are affiliated with the Catholic Church and approx. 26% with the German Evangelical Church while, in Switzerland, approx. 36% are affiliated with the Catholic Church and approx. 24% with the Swiss Reformed Evangelical Church. Other religious traditions are significantly smaller, such as Muslim communities (approx. 5% of the population in each of the two countries), and the free evangelical churches (approx. 1% in Germany and 3% in Switzerland). However, increasingly large groups in both countries are non-denominational (41% in Germany and 29% in Switzerland) (Eurobarometer 2018; Religionswissenschaftlicher Medien- und Informationsdienst e.V (REMID) 2020; Forschungsgruppe Weltanschauungen in Deutschland (FOWID) 2021; Federal Statistical Office 2023). Accordingly, many individuals have a distanced relationship with religion, and many religious organizations have sizeable passive membership.

Environmental organizations have approached religious actors on a national level in both countries, such as WWF 2020 calling on churches for donations,^3^ and Greenpeace supporting a church in installing a photovoltaic system.^4^ There is also increasing collaboration between secular and religious actors and the Swiss Climate Alliance has been joined by various religious organizations such as A Rocha, Fastenaktion, Grüner Fisch, Mission 21, Oeku, Eglise Reformee Vaud and Swiss Quakers.^5^

Religious communities in both countries differ in terms of their recognition under public law (cf. Huber 2022). Such recognition comes with rights such as tax sovereignty (tax collection among members), tax exemption, religious education, hospital and prison chaplaincy, and protection by the state. This legal recognition also comes with obligations such as democratic constitution, respect for the legal system, and representation vis-à-vis the state. In Switzerland, it is the cantonal level that decides on the recognition of religions. The Roman Catholic and Reformed churches are recognized in all cantons, the Christian Catholics (this is a different religion from the Roman Catholic Church) in 10, and the Jewish communities in 6. In Germany, the 16 federal states each decide on recognition within their state. Compared to Switzerland, barriers are lower and it is easier for religious communities to become legally recognized in Germany. Just as in Switzerland, the Protestant and Roman Catholic Churches are legally recognized in all federal states. In some federal states, however, up to 20 different religious organizations are recognized. In Bavaria, for example, humanists, Jehovah’s Witnesses, Christian Science, Pentecostal congregations, New Apostolic communities, and Adventists, are recognized, and in Hesse, Muslim communities and the Baha’I are also recognized.^6^

Accordingly, religious organizations have different positions within society. Therefore, they also differ in terms of the potential they can exploit as well as what may be expected of them.

## Data and methods

To investigate our research questions, we conducted 38 semi-structured interviews in Germany and Switzerland, between 2018 and 2021, using a case-study approach (Yin 2009) focusing on three cities in Germany and one in German-speaking Switzerland. These cities were selected as they are seen as pioneering cities in terms of the energy transition. The social location of interviewees most certainly played a role in shaping their environmental identities, in part because many of the congregations and umbrella organizations interviewed were to some extent actively involved in environmental engagement and were aware of the barriers and tensions to this on a local level.

Interview selection focused on key informants (Chaves et al. 1999) from congregations and umbrella organizations that in most cases held paid positions. These representatives were religious leaders or professionals that know their organization and its environmental activities well, due to their relationship to these activities (e.g., environmental officers in the case of mainline churches).

The interviews covered numerous religious communities, including Catholic, Reformed, Lutheran, Evangelical, other Christian (Old Catholic and Jehovah’s Witnesses), Muslim, Jewish, Buddhist, and Hindu communities (Table 1, below).

**Table 1:** Overview of umbrella organizations and congregations that were interviewed

Unit	Denomination	Country	Interviewee job role	Gender
Congregation (25)	Catholic (5) Reformed (6) Evangelical (7) Other Christian (1) Jewish (2) Muslim (3) Buddhist (1)	Switzerland (8) Germany (17)	Spiritual leader (10) Practical leader (6) Staff (6) Member (1) Key informant* (2)	Female (5) Male (20)
Umbrella organization (13)	Catholic (2) Reformed (2) Ecumenical (1) Evangelical (3) Other Christian (1) Muslim (2) Buddhist (1) Hindu (1)	Switzerland (4) Germany (9)	Manager (8) Environmental officer (4) Politician (1)	Female (1) Male (12)

*Two interviewees did not give their formal job role beyond explaining that they were in paid roles within their organizations

Moreover, we also conducted interviews with representatives of religious umbrella organizations at the national and regional level. It is hard to quantify membership of umbrella organizations as some of them also operate internationally, and did not have regional or national figures (e.g., the Hindu umbrella organization has over 200,000 members internationally), and this is also reflected on a congregational level where active membership is often significantly less than official membership.

Interviews were conducted face-to-face either in situ at the central location (i.e., the church and mosque) for congregations, or at the central office of the umbrella organization within the district. In addition, we gathered information on the training/educational level of interviewees. In terms of training/education, responses not only indicated multiple backgrounds in religion/theology (7 respondents) but also an array of other backgrounds that involve significant training, ranging from journalist, engineer, lawyer, architect, and medical doctor. This gives some indication of the social location of respondents, which could be characterized as vocationally devoted to their religion and mostly favorable to environmental engagement, but with a clear understanding of the resources available to their respective congregations and umbrella organizations.

Of the 38 interviews, 15 were held by two researchers (one male and one female) in which one interviewer conducted the interview whilst the other observed (partially to allow for the honing of interview techniques), with the remainder being held by a single researcher.

The use of semi-structured interviews provided significant advantages: firstly, adherence to a structure ensured a degree of comparability, and secondly, the freedom that is built into semi-structured interviews allowed us to be open and able to react to our interlocutors (Mayring 2002). Interviewees gave their informed consent, and over the course of 60 to 90 min, conversation partners initially explained what their position was within the communities, and then went on to detail how and to what extent their religious communities deal (or do not deal) with environmental issues. At the end of each interview, interviewees were able to make further comments. Interviews were transcribed and coded in MAXQDA. The analysis was carried out partly on the basis of previously fixed theoretical codes (e.g., the four functions of the environmental commitment of religious communities), and partly on the basis of codes that were developed during the analysis. As such, a large part of coding and grouping of codes from the interviews took place following the transcriptions of all interviews and emerged from the data itself which continuously refined and revised our understanding.

In addition to interviews, documents of the religious communities concerning environmental issues were collected and analyzed. These include books, brochures, press releases or the website of religious communities, and also debates in the media. Furthermore, we examined relevant data from the MOSAiCH 2019 project (Stähli et al. 2019).^7^

## Results

### What are religious actors (not) doing to mitigate climate change?

First, we show what religious communities in Germany and Switzerland are (not) doing in the fight against climate change, focusing on the established Christian churches but also including other religious communities (regarding the differences see Huber and Koehrsen 2020; Koehrsen and Huber 2021; Koehrsen et al. 2022). While examining the actions religious communities are taking, we also examine the extent to which this potential is currently fulfilled in the dimensions of A) materialization B) shaping values, and C) political influence.

#### Materialization 

Representatives of different communities explained their commitment by pointing out that climate change is an urgent problem in need of addressing. Communities recognize the problem and now want to act, with some interviewees describing their commitment to the environment as a necessity. In addition, economic reasons were mentioned, as energy efficiency measures often allow for reducing energy costs. If ecological aspects are considered when renovating buildings, this may lead to medium- and long-term cost reductions. Representatives from communities with lower or no environmental engagement also explained the reasons for their low or non-engagement. The main reason given was a lack of resources, both financial and human. One interviewee said:

“*Environmental protection is an issue for the wealthy. If you do not have any financial difficulties, if you know, yes tomorrow I have enough to eat, tomorrow I can pay the electricity in the mosque, tomorrow I can also pay the Imam, that will not be a problem, then you can say: Okay, now we can also dedicate ourselves to this topic. But if you’re always in a state of financial emergency, as is the case with 99 percent of the mosques (…) you just don’t have time to deal with this topic properly now.*” (Interview 5)

Nevertheless, it may be the case that explicit forms of environmentalism are not perceived as taking place, but that implicit pro-environmental attitudes and behaviours take place regardless (Baugh 2019). This may particularly be the case for Muslim communities (such as interview 5, above) and other minority communities in our study. These may indirectly embed an environmental lifestyle due to their smaller economic means in comparison to the established religious communities.

Some interviewees seemed to think that it is simply not possible to engage in climate change mitigation without the appropriate financial resources. Maintenance of the building, payment of employees, etc. are unavoidable, and funds are limited. In addition, the lack of appropriate structures or support was also mentioned.

It was also pointed out that religious communities had other priorities. For example, for many communities, social justice is considered more important, including the social welfare of their members (e.g., welfare services for poor families). Additionally, some communities mentioned migration and discrimination as important issues that they favoured over addressing climate change. Finally, it was also noted that there are other organizations (e.g., civil society organizations) that address the issue of climate change. Accordingly, religious organizations may not feel that they need to do this as well (Interviews 9, 20):

*“But it does not matter. Um, when you come together like that, you obviously have a different goal. The goal of the collaboration is actually, um, to stand together for Christ in this world, right.”* (Interview 9)

As a reason for commitment to climate change mitigation, some interview partners from the established churches pointed out that the churches have to take some responsibility and take action (Interviews 1, 15, and 21). In addition, the potential from available human resources was recognised by some of those interviewed but remains largely untapped. However, we detail below some of the ways in which religious organizations attempt to harness the potential of their membership by promoting environmentally friendly behavior.

An example for the commitment of the established churches in the area of “materialization” is the “Green Rooster.” The “Green Rooster” is an environmental certification scheme for Christian churches for which congregations in Germany and Switzerland can apply. To achieve this certificate, environmental auditors accompany congregations to enable them to reach measurable environmental goals (e.g., saving thermal energy or reducing waste):

*“And once these buildings and all the properties have been recorded, then it’s about making sure that you set yourself small goals, it’s not about completely renovating a parish in a big all-round attack so that it is in a top ecological position. But you can set your own small, ecologically sensible goals.”* (Interview 32)

In addition, interview partners point to various materialization projects in which they are involved. For example, heating, lighting and electrical systems are optimized, heating temperatures lowered, building insulation improved, investments have been made in renewable energy (although in one case a photovoltaic system was not approved due to legislation on the historic preservation of buildings), and palm oil-free cleaning agents have been used. Full-scale commitment to some or many of these activities, however, tends to be the exception. Most congregations undertake more basic activities. For example, respondents reported recycling or buying fair-trade coffee and regional products for their congregational activities. According to an interviewee from a Protestant umbrella organization, there have been some successes, but there is also significant potential for further engagement.

“*When I see that we have been able to successfully introduce energy management systems in many of our regional churches and have thus far exceeded our 2015 climate targets of saving 25% CO*_*2*_* in buildings, then that’s great. But I see that there is still a lot we can do in the areas of procurement and food, and that this will lower our overall targets again. Mobility is an issue where I think we could also make progress.*” (Interview 24)

Religious actors engage in the fight against climate change in various forms. However, it is still only a minority of communities that are highly engaged in different types of materialization activities (e.g., reducing temperature, using photovoltaics, and improved building insulation). Most of those that are engaged often do so on a low level (e.g., buying fair-trade coffee). While many consider it important to address climate change, they are unable and/or unwilling to invest more.

#### Shaping values

In this area, the religious communities interviewed are active in various forms. Representatives of all Abrahamic religions mentioned, for example, that the Holy Scriptures already include environmental protection, and thus climate change mitigation. If adherents live their life according to the scriptures, they should automatically be environmentally friendly. While representatives of Jewish and Muslim communities (e.g., interviews 14 and 16) concluded that this was enough, others (evangelicals) stated that this was what they were trying to communicate to their followers—however, they point out that it is ultimately up to individuals to decide how they want to live (interviews 6 and 12):

*“So, of course we have, but of course that’s not the core task of the church, to somehow pass this on to all members. So, I feel like that might be going a little (too) far.”* (Interview 20)

Many communities were more active in shaping the values of the adherents. Climate change is addressed in individual sermons or is part of an environmental education within the communities. Further, various religious communities produce or use magazines or brochures to educate their members. Christian and Muslim umbrella organizations provide material for this purpose (for Muslim environmental activism in Germany, see also Willms 2021). For instance, in Switzerland, the association “Oeku Church and Environment” produces publications, such as the environmental handbook “*Let’s go green*” and guidelines for saving energy. Also, the Muslim organization VIOZ (Association of Islamic Organizations in Zurich) published an environmental brochure “*Environmental Protection and Sustainability in Islam*.” The major Christian churches often emphasize that the environment is important in youth education. Climate change is discussed within the Scouts or at youth camps. Thus, attempts are also being made by different means and to different extents to bring climate protection and appropriate behavior closer to the followers. This task should not be underestimated, as one interviewee says:

“*And that is actually quite a lot of educational work, but I have the feeling that there should be more, it should bare more fruit, when this is really implemented. However, of course, there are many people and to reach all of them and … I have the feeling that (this) is such an ongoing task, to just stick to it. That you make people aware of this again and again.*” (Interview 20)

Even though many of those interviewed noted that climate change is addressed within the community, the issue tends to remain in the background. However, some recognized that more should be done in this regard.

**Graph 1:**
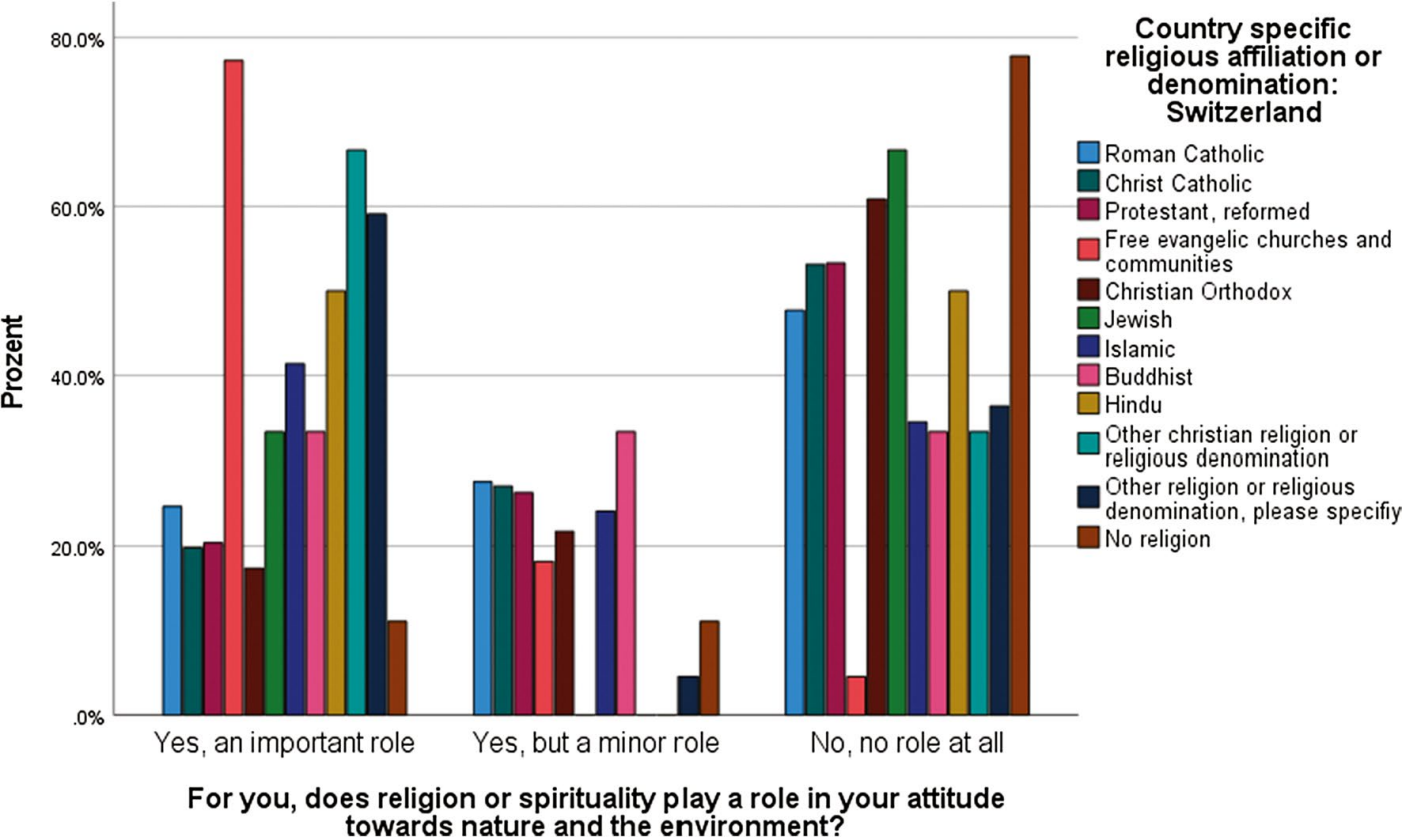
Authors own elaboration, data taken from MOSAiCH, 2019

As a Swiss survey shows (Stähli et al. 2019), respondents believed that their attitudes toward nature and the environment are affected by religion. Of those surveyed, 15.1% said religion played an important role in attitudes toward nature and the environment; 14.9% said it played a role, but not as important; and 42.7% said none at all. 27.3% gave no answer. In effect, of those that responded, more than 40% indicated that they believed that religion plays a role in shaping attitudes and behaviors towards the environment. Furthermore, the potential appears to be smaller for communities that address climate change than for those that do not or rarely address it. Graph 1 shows that adherents of both the Roman Catholic Church and the Reformed Church are less likely to state that religion plays an important role in attitudes toward nature and the environment than adherents from other religious groups. Indeed, the followers of evangelical free churches attribute an important role to religion in this area, with almost 80% of the surveyed followers of evangelical communities claiming this is important. Other Christian communities also show high values in this respect. This can probably be attributed to the fact that religion seems to play a central role in the lives of adherents from these Christian denominations and that Christian identity is perceived as a determining factor in all other areas (Stolz and Huber 2014). Non-Christian religions, including Islam, Judaism, Hinduism, and Buddhism also show relatively high values. Here, too, there is still hidden potential. This can often be attributed to the fact that in migrant communities’ religion forms an important part of identity, and strongly influences life (Stolz et al. 2014). The great potential of religion for addressing climate change appears to ultimately lay in the transmission of values. However, religion is but one of many ways that values are transmitted. It may be that adherents to different faiths perceive other factors as being more important for informing their environmental values (e.g., education, mass media consumption, and family members). Furthermore, values are not transmitted uniformly, and there are significant tensions that can be identified within denominations, between denominations, between religions, and between a religion and wider society (Koehrsen et al. 2022).

#### Political influence

Political influence offers a potential that is acquired through power. Here, however, the question arises to what extent attempts are made to use it. In Switzerland and Germany, the established Christian churches have a certain amount of political influence via their public visibility and social ties with political parties and decision-makers. Other religious traditions only have a marginal influence at best. The focus of the established Christian churches is usually on social issues or justice, and not on climate change. At the level of local congregations, political involvement is sporadic, although individual churches get involved in voting or elections. One example is the Swiss popular initiative for responsible businesses.^8^ This initiative sought to create a new law for Swiss enterprises to follow human rights and environmental standards in their economic activities abroad. Various religious organizations (mainly related to the established churches) joined in the initiatives committee,^9^ producing and displaying posters and distributing flyers. The public debate on this initiative was very controversial, and it should be noted that the main focus was on justice; with environmental protection and climate change forming only a minor part of this. Religious engagement in such political matters as the popular initiative for responsible businesses is often coordinated by umbrella organizations rather than at a local level. An explicit example of lobbying is shown on the occasion of the climate conference in Katowice (COP24): In a joint letter, the Swiss Bishops’ Conference, the Christian Catholic Church of Switzerland, and the Federation of Swiss Protestant Churches appealed to the Federal Council to advocate for a fairer climate policy. Umbrella organizations have often made attempts to influence politics:

*“I participate in various working groups in the Bundestag- There is also a working group `Peace Responsibility of the Religions`, that I am a part of.”* (Interview 29)

Interviewees themselves also see the potential of religious activism in this area. It is possible to approach politicians from a Christian perspective, especially those from overtly Christian parties (such as the CVP, CDU). Thus, there is also the possibility to work on climate protection as a topic from a non-left perspective (Interviews 1 and 21). Recently, various actions have also taken place on the local level. Particularly in the context of *Fridays for Future*, some Christian churches have provided climate strikers with rooms, prayed for them, and organized panel discussions, even creating a network called “Churches for Future”.^10^

### How do religious actors deal with expectations?

Interviewees spoke of various expectations which they felt were placed on religious communities regarding climate change. Often, it was these very expectations that resulted in galvanizing communities to take action against climate change. First, umbrella organizations have certain expectations with regard to individual congregations. Then, there are also expectations from the congregations’ own members, and finally those, which are perceived as coming from outside of the community. However, these cannot always be clearly demarcated.

#### Expectations from umbrella organizations or leaders of one’s own religious tradition

Various interviewees referred to their own religious tradition in their commitment to combating climate change. From the Christian perspective, the best-known example of this is the *Laudato Si’* encyclical which focuses on the preservation of creation. Various interviewees from Christian churches claimed their commitment was based on theology or, more specifically, on *Laudato Si’* (Interviews 12, 19). Very involved individuals noted with regret that they had hoped for more change within the churches from *Laudato Si’*. One person from a Reformed umbrella organization pointed this out:

*“Although they [the Catholic Church] have actually received the boost with Laudato Si’, very little has actually happened so far.”* (Interview 38).

To date, changes have failed to materialize, partially because of resistance from within churches (Interviews 1 and 10).

Non-Christian religious actors also face expectations within their own religious tradition: Muslim representatives mentioned that sustainability is a major theme in the Quran (Interviews 10 and 13), and Jewish interviewees referred to the Torah and God (Interview 16). Buddhists argued that although it was not an issue directly, it was implicit in the Buddha’s teachings (Interview 8) and a representative of a Hindu community said that care of the environment is also important in the Hindu faith (Interview 22). Thus, representatives of all religious traditions pointed out that their own religion expected its followers to care for the environment and the climate. However, this is rarely a top priority.

#### Expectations from members

Reference was also made to the congregations’ own members who have brought environmental commitment into the given congregations. While some representatives of non-Christian communities claimed that climate change does not interest their members, representatives of mainstream Christian churches noted that they also had many environmentally committed members, who had originally engaged outside of the community in environmental activities. Over time, however, they have brought this commitment into the community. This is also reflected to some extent in non-Christian communities, as can be seen in the quote below where younger members were the initiators of a project to create an environmentally friendly mosque:

“…*green mosque has become a very attractive topic. And especially (for) young people, young Muslims. The first generation was very satisfied if a room was found for prayer. Whether that's in a garage or in the backyard or somewhere else, but now Muslims - especially the new generation - pay attention that their mosques are representative and must be environmentally friendly. Green mosques that are ecological in how they are built, with the way they are operated...There are many features that this Green Mosque does with energy. That energy can be obtained from these new energy sources in the most environmentally friendly way possible."* (Interview 13)

This shows that members of religious communities can be drivers of religious engagement in climate change mitigation. It was also often emphasized that it was important for members to be actively involved in order to be able to achieve something and that volunteers are required to attain success.

#### External expectations 

Interviewees also talked about how commitment was contextually driven from society, with, for example references made to *Fridays for Future*. Climate strikers asked communities to provide them with space and other forms of support. In some communities, “greening” activities were initiated directly as a result of this, particularly value dissemination in the form of green themed sermons. In the words of one interviewee: *“Fridays for Future and Greta Thunberg, have brought a certain movement into the running”* (Interview 29). However, other environmental organizations have also brought the issue to the church:

*“...Ecology didn’t grow on the church’s dung heap, that’s quite clear. There have been other groups. There was WWF, Greenpeace, etc., which really had a pioneering role in this area. (...) Then they also found partners in churches, which supported them afterwards, or then they founded their own organizations, which repeatedly tried to raise awareness in the churches.”* (Interview 12)

The established Christian churches in particular pointed out that they see themselves as a reflection of society and take up these trends accordingly. If climate change is becoming more and more important in society, and if it is also a defining issue for the public and in politics, the churches cannot abstain from action. Another formulated this in more general terms:

*“Especially in the last elections to the National Council, where there was a green landslide—I’ll say that—plus the whole issue of the “Fridays for future,” the topic has once again been put a little more prominently on the agenda. It had been a side issue for a long time [...] and now, because of its socio-political relevance, it has taken on a different significance [...] and that’s why you have to deal with this topic in a different way.”* (Interview 38)

Different expectations are perceived and addressed in religious communities, but reactions to these expectations vary. This may lead to increased commitment, trend-setting, or counter-movements. Interestingly, communities that do not see themselves as a reflection of society are better able to set aside expectations or to directly reject them. A representative of an evangelical community, for example, said:

*“I think that churches don’t primarily have an ecological mission, but I sometimes have the impression that we miss the actual mission a bit. To be able to love people from faith somewhere. And to see more in people than just ecology.*” (Interview 25)

This was echoed by an interviewee from the Buddhist community:

“*It is not the task of Buddhist communities to educate people about environmental protection. That is not the task. The task of Buddhism is the training of the spirit. I mean a sports or football club does not have the task to enlighten about environmental protection either.”* (Interview 2)

## Discussion

Our research shows that there were no significant differences between stakeholders interviewed in Germany and Switzerland, echoing the notion that there are strong structural similarities between the two (Koehrsen and Huber 2021). Whether this means that our findings could be interpreted as valid for the entire German speaking part of Europe is another matter. But the similarities in responses across both countries certainly indicate that there may be wider-reaching implications of our research.

Many of the umbrella organizations and congregations interviewed were active in communicating environmentally friendly values to some extent. However, there were differences in the extent to which these values are communicated and in the extent to which it is considered useful to do so at all. While the topic of climate change is considered as important in the two large Christian churches, it plays only a subordinate role in other religious denominations. This may partially be owing to the social location of other religious denominations which are not officially established and thus do not have access to the funding available to the two established churches (raised via a church tax). However, there is no added financial burden to preaching environmentally friendly behaviour, which may indicate that there are other reasons for this lack of engagement in communicating environmentally friendly behaviour. One possible explanation for this is that smaller religious groups (e.g., Muslim congregations) often comprise of higher numbers of migrants who may be socially integrated but who are excluded from official participation in both Swiss political processes (such are referendums) and national political processes in Germany. There is great potential here for further engagement (Grim and Tucker 2014; Haluza‐DeLay 2014; Koehrsen 2015, 2018; Jenkins et al. 2018; Ives and Kidwell 2019), but this has not been fully harnessed. This further strengthens previous research which indicates that although some action is taking place in addressing climate change, this is not currently the main concern of religious organizations (Caldwell et al. 2022).

Religion can have a moral role model function and close social ties to its members. Looking at the empirical data, it appears that while the larger established Christian churches are the most active, the potential for change lies primarily with Muslim and Evangelical congregations. In the case of Muslim congregations, as mentioned above, this potential may be affected by the fact that a large proportion of members are migrants that may have more limited financial means. Since religion is often a first point of contact (Norris and Inglehart 2004), there is the possibility of conveying corresponding values based on a religious rationale. Furthermore, despite their reputation as highly developed wealthy countries, there are considerable levels of energy poverty in both Germany and Switzerland that may further increase in the course of the ongoing energy transition processes (Hearn et al. 2022). Therefore, a potential measure that addresses both, climate change and social justice, could be religious campaigning persuading political decision makers to channel funds towards those that are most vulnerable.

The potential for Evangelical congregations stems above all from the fact that Evangelicals tend to show higher participation rates in congregational activities (e.g., church services). Therefore, religion has a higher chance to penetrate other areas of members’ everyday life. Thus, evangelicals may also draw on religious principles in their attitudes toward the economy, politics, and even the environment (Stähli et al. 2019). If environmentally friendly attitudes become part of evangelical culture, they can spread throughout the evangelical milieu and lead to a change of lifestyle among the adherents (e.g., choices about energy consumption and mobility). It should be noted that although there is some research to indicate that members of monotheistic religions are currently less likely to engage in pro-environmental behaviour than members of pantheistic religions (Taylor et al. 2020), as the climate crisis deepens, this may well change, and the potential reach of monotheistic religions within a European context is significant.

Globally, there have been some attempts from religious organizations to exert *political influence*, such as the Evangelical Climate Initiative in the USA (Wardekker et al. 2009; Johnston 2010). In this respect, individual attempts can be positively highlighted, but the extent to which the potential for further action exists here is less visible, and would require a deeper analysis of the relationship between state and religions. Furthermore, the case could be made for umbrella organizations acting as mediators between congregations and politics, providing the contacts and necessary networks to influence both political decisions and the uptake of policies by congregations.

In the area of *materialization*, some religious communities engage directly in climate change mitigation. They reduce CO_2_ emissions by decreasing their energy consumption and improving energy efficiency of their buildings, or through producing their own electricity renewably. The implementation of concrete projects can indeed make a difference locally but when compared to other social spheres (e.g., business sector and especially industrial production) the potential contribution of religious organizations appears small. Nevertheless, one could argue that religious organizations could use their moral authority and act as role models for others when they undertake materialization measures, thereby encouraging others to follow, creating a much broader impact (Mohamad et al. 2012).

In terms of *resources*, these are often perceived as an essential prerequisite for climate change mitigation (Gardner 2003, 2006; Haluza‐DeLay 2014), either in the form of financial or human resources. Indeed, our empirical work revealed that the lack of financial resources was perceived of as a significant obstacle.

However, the increasingly visible financial benefits of some forms of materialization (such as installing PV) help to make a financial case for using limited resources on environmentally friendly measures with investments in PV (Martinopoulos 2020). Furthermore, the significant human capital that is available to congregations in terms of membership provides the potential for significant change without necessitating financial input. Thus, for example, congregations can be encouraged to take actions such as reduce meat consumption and recycle more, through the use of environmentally friendly sermons which do not have a cost attached to them. In the context of the Catholic Church, these kinds of measures also add clarity to the *Laudato Si’* encyclical, providing concrete ways of addressing environmental issues (Wilkins 2020).

The interviewees were also aware of their role within society and what expectations they face regarding climate change mitigation. In both countries, established Christian churches see themselves as an integral part of society and try to take up corresponding trends and to participate in the fight against climate change. This may well be connected to recognition and perceived potential from the general public. Non-established religious organizations, on the other hand, were able to opt out in this regard, as they did not face such expectations from society, environmental organizations, or their own members. Finally, with regard to expectations, it can be noted that religious actors may believe that potentials need not be exhausted, and indeed, it may be unrealistic to expect them to take the lead in climate change mitigation. They point out that their core purpose is spiritual, and that there are other organizations that are specifically designated to reducing the impact of climate change. However, if congregations take up a greater role in mitigating climate change following encouragement from leadership, the potential for significant change could be considerably amplified.

In terms of engagement, the national contexts also need to be considered. In the case of Switzerland, current temperatures have risen considerably faster than the global average (NCCS 2023), with a noticeable and visible to the eye effect on glacier decline (10% decrease in the past 2 years (Horton and reporter 2023; GLAMOS 2023)). In addition, the impact of social movements such as Fridays for Future have been substantial with a recent study indicating that environmental concern and behaviour have been positively influenced with behavior changes reported even among those that are sceptical of the climate change movement (Fritz et al. 2023). In the case of Germany, the catastrophic flooding in 2021 had a noticeable effect on votes in favor of climate progressive parties (Garside and Zhai 2022), and the Green Party’s share of votes increased from 8.8% in 2017 to 14.8% in 2021 indicating broader societal tendencies towards increased environmentalism. In light of this, religious environmental engagement in both countries appears to be relatively low (in comparison to the broader societal engagement in the two countries), leading us to infer that much of the assumed potential of religion remains as yet unrealized.

It is important to note that there are limitations to our research which we have tried to address. Although we conducted numerous interviews, the professional role of each interviewee in the given organization may have had an impact on how they framed their responses. Additionally, our research focused on the cases of Germany and Switzerland. The dynamics found here may be to some extent similar in other Western European countries. However, our results do not necessarily translate well to countries outside of Europe, and specifically the Global South, where GHG emissions are overall significantly lower. However, the Global South is not a singular monolithic entity as the distribution of GHG emissions is heavily concentrated in emerging economies such as India, Brazil, and China, with 10 countries accounting for some 78% of Global South emissions, while the remaining 120 countries account for only 22%. (Fuhr 2021), and the case could be made for examining such emerging economies separately.

## Outlook

With regard to future research, the Global South and emerging economies are certainly important, where attitudes on climate change may be more influenced by religion. In less secularized environments, religion has more impact and can (at least theoretically) better develop the potentials discussed in this article. However, financial resources are often sparser in the Global South, which in turn weakens the potential of climate-mitigating action. It is also important to weigh up where the greatest savings potential lies in terms of solving the climate crisis. For example, countries in the Global North have a much larger per capita CO_2_ footprint than those in the Global South. At the same time, due to economic development, countries in the Global South may move towards a similar footprint as the Global North. This is a major challenge that could possibly be mitigated by religion and an interesting field for future research.

Further research could also focus on differences in engagement between religious umbrella organizations and congregations and to what extent umbrella organizations inhibit engagement at the congregational level because there may be a perception that this topic is already being addressed. This may mean that umbrella organizations act as both inhibitors *and* facilitators of engagement in addressing climate change. Moreover, an interesting avenue for future research would be to study congregations and umbrella organization that actively support environmentally harmful human practices and industries (e.g., mining industry and non-renewable energies).

This article has shown the potentials attributed to religion in the fight against climate change, with various expectations placed on religion by scientific, social, and religious actors. Based on empirical data from Germany and Switzerland, we show that many of these expectations remain, so far, unfulfilled. There appears to be significant untapped potential. Finally, the question also arises: Do the expectations have to be fulfilled at all? If one looks at other areas in society—such as the political and economic sphere—some may argue that these other areas do not contribute as much as they could to address climate change. In contrast to religious actors, other actors may wield greater decision-making power and may have higher greenhouse gas emissions to account for. Moreover, one may question whether religion should be seen as set of resources to solve the climate crisis. Is it the task of religious communities to address environmental issues or should these rather be addressed by other types of actors (e.g., politicians and business leaders)? However, in addition to the external demands, there are internal demands and all major religious traditions now have guidelines that call for action against climate change. To what extent these will be followed through is yet unclear.

## Data Availability

The datasets generated during and/or analyzed during the current study are not publicly available due to privacy restrictions.

## Footnotes

1 See sidebar at: https://fore.yale.edu/World-Religions

2 http://www.hinduclimatedeclaration2015.org/english

3 https://www.wwf.de/aktiv-werden/als-gemeinschaft-sammeln/kirchengemeinde

4 https://www.greenpeace.ch/de/story/10467/sonnenenergie-vom-kirchendach

5 https://www.klima-allianz.ch/mitglieder/

6 https://www.personenstandsrecht.de/Webs/PERS/DE/informationen/religionsgemeinschaften/religionsgemeinschaften-node.html).

7 The data is available at: https://devisu.forscenter.ch/index.php/catalog/1120

8 https://www.admin.ch/gov/en/start/documentation/votes/20201129/iniziativa-popolare-per-imprese-responsabili-a-tutela-dell-essere-umano-e-dell-ambiente.html

9 See: https://konzernverantwortung.ch/koalition/

10 Churches for Future-Kirchen für Klimagerechtigkeit (kirchen-fuer-klimagerechtigkeit.de).

## References

[CR1] Arbuckle MB (2017). The interaction of religion, political ideology, and concern about climate change in the United States. Soc Nat Resour.

[CR2] Barker DC, Bearce DH (2013). End-times theology, the shadow of the future, and public resistance to addressing global climate change. Polit Res Q.

[CR3] Baugh AJ (2017). God and the green divide: religious environmentalism in black and white.

[CR4] Baugh AJ (2019). Explicit and embedded environmentalism: challenging normativities in the greening of religion. Worldviews: Global Relig Cult Ecol.

[CR5] Baumann M, Stolz J (2007) Eine Schweiz-viele Religionen: Risiken und Chancen des Zusammenlebens. transcript Verlag, 2007: Chapter Religiöse Vielfalt in der Schweiz: Zahlen, Fakten, Trends, pp 39–66

[CR6] Becci I (2022) Grounding eco-spiritualities: insights drawing on research in Switzerland. AЯGOS. 10.26034/fr.argos.2022.3558

[CR7] Bergmann S (2009). Climate change changes religion. Stud Theol Nord J Theol.

[CR8] Berry E (2022) The right climate, p 122. In: Berry E (ed) Climate politics and the power of religion

[CR9] Biel A, Nilsson A (2005). Religious values and environmental concern: harmony and detachment*. Soc Sci Q.

[CR10] Bjork-James S (2023). Lifeboat theology: white evangelicalism, apocalyptic chronotopes, and environmental politics. Ethnos.

[CR11] Blanc J (2017) Ökokatholizismus: sozialethische Untersuchungen zu ausgewählten Ländern und Institutionen in Europa. In: Beiträge zur sozialwissenschaftlichen Nachhaltigkeitsforschung Band 21. 1. Auflage. Metropolis-Verlag, Marburg

[CR12] Blanc J (2022) From “why should?” to “why do?” tensions in the Christian context while acting for the environment 1. In: Koehrsen J, Blanc J, Huber F (eds) Religious environmental activism: Emerging conflicts and tensions in earth stewardship. Taylor & Francis, Routledge, pp 112–131

[CR13] Bochinger C, Baumann M, Becci I, Mader L, Pahud de Mortanges R, Schinzel M, Stolz J, Frank K (eds.) (2012) Religionen, Staat und Gesellschaft: die Schweiz zwischen Säkularisierung und religiöser Vielfalt

[CR14] Boff L (2011) Ecología: grito de la Tierra, grito de los pobres, 5th edn. Editorial Trotta, S.A, Publisher

[CR15] Buckley DT (2022). Religious elite cues, internal division, and the impact of Pope Francis Laudato Si’’. Polit Relig.

[CR16] Caldwell C, Probstein N, Yoreh T (2022). Shades of green: environmental action in places of worship. J Environ Stud Sci.

[CR17] Carr W, Patterson M, Yung L, Spencer D (2012). The faithful skeptics: evangelical religious beliefs and perceptions of climate change. J Study Religion Nat Cult.

[CR18] Casanova J (1994). Public religions in the modern world.

[CR19] Chaplin J (2016). The global greening of religion. Palgrave Commun.

[CR20] Chaves M (2004). Congregations in America.

[CR21] Chaves M, Konieczny ME, Beyerlein K, Barman E (1999). The national congregations study: background, methods, and selected results. J Sci Study Relig.

[CR22] Christie I, Gunton RM, Hejnowicz AP (2019). Sustainability and the common good: Catholic social teaching and “integral ecology” as contributions to a framework of social values for sustainability transitions. Sustain Sci.

[CR23] Clingerman F, O’Brien KJ (2017). Is climate change a new kind of problem? The role of theology and imagination in climate ethics. Wires Clim Change.

[CR24] Danielsen S, DiLeo DR, Burke EE (2021). US Catholic bishops’ silence and denialism on climate change. Environ Res Lett.

[CR25] Dasgupta P, Ramanathan V (2014). Environment and development Pursuit of the common good. Science (New York, N.Y.).

[CR26] Dessì U (2015). Japanese religions and globalization.

[CR27] Djupe PA, Gwiasda GW (2010). Evangelizing the environment: decision process effects in political persuasion. J Sci Study Relig.

[CR28] Djupe PA, Hunt PK (2009). Beyond the Lynn White thesis: congregational effects on environmental concern. J Sci Study Relig.

[CR29] Ecklund EH, Scheitle CP, Peifer J, Bolger D (2017). Examining links between religion, evolution views, and climate change skepticism. Environ Behav.

[CR30] Eurobarometer (2018) *Soziale situation in Deutschland: religion*. [Online] [online]. Available from: https://www.bpb.de/kurz-knapp/zahlen-und-fakten/soziale-situation-in-deutschland/145148/religion/ (Accessed 26 April 2023).

[CR31] Federal Statistical Office (2023) Religions. [Online] [online]. Available from: https://www.bfs.admin.ch/bfs/en/home/statistiken/bevoelkerung/sprachen-religionen/religionen.html (Accessed 27 April 2023).

[CR32] Foltz R (2006). Nature in Asian traditions: the state of the field. Worldviews: Glob Relig Cult Ecol.

[CR33] Forschungsgruppe Weltanschauungen in Deutschlan (FOWID) (2021) Anthroposophen und Nicht-Geimpfte. [Online] [online]. Available from: https://fowid.de/meldung/anthroposophen-und-nicht-geimpfte (Accessed 26 April 2023).

[CR34] Fossey K (2003) Buddhist faith statement. In: Alliance of religions and conservation

[CR35] Francis P (2015). Encyclical letter Laudato si’ of the Holy Father Francis.

[CR36] Fritz L, Hansmann R, Dalimier B, Binder CR (2023). Perceived impacts of the Fridays for future climate movement on environmental concern and behaviour in Switzerland. Sustain Sci.

[CR37] Fuhr H (2021). The rise of the Global South and the rise in carbon emissions. Third World Q.

[CR38] Gade AM (2019) Google-Books-ID: XSd5DwAAQBAJ. Muslim environmentalisms: religious and social foundations. Columbia University Press

[CR39] Gade AM (2023). Muslim environmentalisms and environmental ethics: theory and practice for rights and justice. Muslim World.

[CR40] Gardner G (2003) Engaging religion in the quest for a sustainable world. In: State of the world 2003, 20th edn. Routledge. 10.4324/9781849776257-15

[CR41] Gardner GT (2006) Inspiring progress: religions’ contributions to sustainable development. In: ‘A worldwatch book’--Cover. W.W. Norton, New York

[CR42] Garside S, Zhai H (2022). If not now, when? Climate disaster and the Green vote following the 2021 Germany floods. Res Polit.

[CR43] Glaab K (2022) The green, the secular, and the religious: the legitimacy of religious environmentalism in global climate politics. In: Koehrsen J, Blanc J, Huber F (eds) Religious Environmental Activism: Emerging Conflicts and Tensions in Earth Stewardship. Taylor & Francis, Routledge, pp 268–281

[CR44] GLAMOS (2023) Swiss Glaciers | Glacier monitoring in Switzerland GLAMOS. [online]. Available from: https://www.glamos.ch/en/. Accessed 3 Oct 2023

[CR45] Gottlieb RS (2006). The Oxford handbook of religion and ecology.

[CR46] Gottlieb R (2008). ‘You gonna be here long? Religion and sustainability. Worldviews: Glob Relig Cult Ecol.

[CR47] Greeley A (1993). Religion and attitudes toward the environment. J Sci Study Relig.

[CR48] Grim J, Tucker ME (2014). Ecology and religion.

[CR49] Gutsche G (2019). Individual and regional Christian religion and the consideration of sustainable criteria in consumption and investment decisions: an exploratory econometric analysis. J Bus Ethics.

[CR50] Haluza-DeLay R (2014). Religion and climate change: varieties in viewpoints and practices. Wires Clim Change.

[CR51] Harmannij D (2019) In: Filho WL, McCrea AC (eds) Is it possible to give environmental issues a more prominent role in church life? pp 97–114. 10.1007/978-3-319-95336-6_6

[CR52] Harmannij D (2022) Environmental action within local faith communities: navigating between high expectations and practical action. In: Koehrsen J, Blanc J, Huber F (eds) Religious environmental activism: emerging conflicts and tensions in earth stewardship. Taylor & Francis

[CR53] Harris I (1995). Buddhist environmental ethics and detraditionalization: the case of EcoBuddhism. Religion.

[CR54] Hayhoe K (2019) I’m a climate scientist who believes in God. In: Hear me out. The New York Times. https://www.nytimes.com/2019/10/31/opinion/sunday/climate-changeevangelical-christian.html. Accessed 27 Dec 2023

[CR55] Hearn AX, Mihailova D, Schubert I, Sohre A (2022) Redefining energy vulnerability, considering the future. Front Sustain Cities 4:952034. 10.3389/frsc.2022.952034

[CR56] Hempel L, Smith EK (2020). Evangelical protestantism, politics, and the environment: when and how do biblical beliefs matter?. Soc Nat Resour.

[CR57] Hitzhusen GE, Tucker ME (2013). The potential of religion for Earth Stewardship. Front Ecol Environ.

[CR58] Hoffman JIE (2015) Chapter 13 - Hypergeometric distribution. In: Biostatistics for medical and biomedical practitioners. Academic Press, pp 179–182. 10.1016/C2014-0-02732-3

[CR59] Horton H, Reporter HHE (2023) Swiss glaciers lose 10% of their volume in two years. The Guardian. https://www.theguardian.com/environment/2023/sep/28/swiss-glaciers-lose-tenth-volume-in-two-years-climate-crisis. Accessed 27 Dec 2023

[CR60] Huber F (2022) Environmentalism in the religious field: the role of the establishment for competition in Switzerland, Routledge. pp 157–175. In: Koehrsen J, Blanc J, Huber F (eds) Religious environmental activism: Emerging conflicts and tensions in earth stewardship. Taylor & Francis

[CR61] Huber F, Koehrsen J (2020) Das Ergrünen von Religionen: Ökologische Nachhaltigkeit in religiösen Gemeinschaften. In: Henkel A, Barth T (eds) 10 Minuten Soziologie: Nachhaltigkeit. transcript, Bielefeld. 10.14361/9783839449684-009

[CR62] Hulme M (2017). Climate change and the significance of religion. Econ Pol Wkly.

[CR63] International Climate Change Symposium (2015) The Islamic declaration on global climate change. https://unfccc.int/news/islamic-declaration-on-climate-change. Accessed 27 Dec 2023

[CR64] IPCC (ed.) (2022) Climate change 2022: impacts, adaptation and vulnerability. In: Contribution of Working Group III to the Sixth Assessment Report of the Intergovernmental Panel on Climate Change. https://www.ipcc.ch/report/ar6/wg2. Accessed 27 Dec 2023

[CR65] Ives CD, Kidwell J (2019). Religion and social values for sustainability. Sustain Sci.

[CR66] Jenkins W, Berry E, Kreider LB (2018). Religion and climate change. Annu Rev Environ Resour.

[CR67] Johnston L (2010). The religious dimensions of sustainability: institutional religions, civil society, and international politics since the turn of the twentieth century. Relig Compass.

[CR68] Kanagy CL, Nelsen HM (1995). Religion and environmental concern: challenging the dominant assumptions. Rev Relig Res.

[CR69] Kanagy CL, Willits FK (1993). A “greening” of religion? Some evidence from a Pennsylvania sample. Soc Sci Q.

[CR70] Koehrsen J (2015). Does religion promote environmental sustainability? – exploring the role of religion in local energy transitions. Soc Compass.

[CR71] Koehrsen J (2018). Religious agency in sustainability transitions: between experimentation, upscaling, and regime support. Environ Innov Soc Trans.

[CR72] Koehrsen J, Huber F (2021). A field perspective on sustainability transitions: the case of religious organizations. Environ Innov Soc Trans.

[CR73] Koehrsen J, Blanc J, Huber F (2022). How “green” can religions be? Tensions about religious environmentalism. Z Relig Ges Polit.

[CR74] Koehrsen J, Blanc J, Huber F (2023). Religious environmental activism: emerging conflicts and tensions in Earth stewardship.

[CR75] Landrum AR, Lull RB, Akin H, Hasell A, Jamieson KH (2017). Processing the papal encyclical through perceptual filters: Pope Francis, identity-protective cognition, and climate change concern. Cognition.

[CR76] Lawson SL, Ramey SW (2018). Sourcing stereotypes: constructing and challenging simplified knowledge. Cult Relig.

[CR77] Li N, Hilgard J, Scheufele DA, Winneg KM, Jamieson KH (2016). Cross-pressuring conservative Catholics? Effects of Pope Francis’ encyclical on the U.S. public opinion on climate change. Clim Change.

[CR78] Maibach E, Leiserowitz A, Roser-Renouf C, Myers T, Rosenthal S, Feinberg G (2015). The Francis effect: how Pope Francis changed the conversation about global warming.

[CR79] Martinopoulos G (2020). Are rooftop photovoltaic systems a sustainable solution for Europe? A life cycle impact assessment and cost analysis. Appl Energy.

[CR80] Mayring P (2002) Einführung in die qualitative Sozialforschung: eine Anleitung zu qualitativem Denken. Studium Paedagogik. 5., überarbeitete und neu ausgestattete Auflage. Weinheim ; Basel: Beltz Verlag

[CR81] McKibben B (2001). Where do we go from here?. Daedalus.

[CR82] Mohamad ZF, Idris N, Mamat Z (2012). Role of religious communities in enhancing transition experiments: a localised strategy for sustainable solid waste management in Malaysia. Sustain Sci.

[CR83] Myers TA, Roser-Renouf C, Maibach E, Leiserowitz A (2017). Exposure to the Pope’s climate change message activated convinced Americans to take certain activism actions. Glob Challenges (hoboken, NJ).

[CR84] NCCS (2023) *Observed climate change in Switzerland*. [Online] [online]. Available from: https://www.nccs.admin.ch/nccs/en/home/climate-change-and-impacts/swiss-climate-change-scenarios/observed-climate-change-in-switzerland.html (Accessed 3 October 2023).

[CR85] Norris P, Inglehart R (2004). Sacred and secular: religion and politics worldwide.

[CR86] Pew Research Center (2017) The changing global religious landscape. https://www.pewresearch.org/religion/2017/04/05/the-changing-global-religious-landscape/. Accessed 27 Dec 2023

[CR87] Pickel G (2011). Religionssoziologie.

[CR88] Pollack D, Olson DVA (eds) (2007) The role of religion in modern societies, 1st edn, Routledge. 10.4324/9780203942239

[CR89] Posas P (2007) Roles of religion and ethics in addressing climate change. Ethics Sci Environ Polit 2007:31–49. 10.3354/esep00080

[CR90] Preston JL, Baimel A (2021). Towards a psychology of religion and the environment. Curr Opin Psychol.

[CR91] Ramey S (2007). Challenging definitions: human agency, diverse religious practices and the problems of boundaries. Numen.

[CR92] Religionswissenschaftlicher Medien- und Informationsdienst e.V (REMID) (2020) Religionen & Weltanschauungsgemeinschaften in Deutschland: Mitgliederzahlen – REMID – Religionswissenschaftlicher Medien- und Informationsdienst e.V. https://www.remid.de/. Accessed 27 Dec 2023

[CR93] Saniotis A (2012). Muslims and ecology: fostering Islamic environmental ethics. Contemp Islam: Dyn Muslim Life.

[CR94] Schuldt JP, Pearson AR, Romero-Canyas R, Larson-Konar D (2017). Brief exposure to Pope Francis heightens moral beliefs about climate change. Clim Change.

[CR95] Sherkat DE, Ellison CG (2007). Structuring the religion-environment connection: identifying religious influences on environmental concern and activism. J Sci Study Relig.

[CR96] Shibley MA, Wiggins JL (1997). The greening of mainline american religion: a sociological analysis of the environmental ethics of the national religious partnership for the environment. Social Compass.

[CR97] Short HE (1985) Gathered for life: Official report, VI assembly, World Council of Churches, Vancouver, Canada, 24 July-10 August, 1983. Edited by David Gill. Grand Rapids, Wm. B. Eerdmans Publishing Co., 1983. viii 355 pp. $12.95. Church History 54(2):285-285. 10.2307/3167312

[CR98] Stähli ME, Sapin M, Pollien A, Ochsner M, Nisple K (2019) MOSAiCH 2019. Measurement and observation of social attitudes in Switzerland. In: Study on social inequality and related topics. Lausanne: Swiss Centre of Expertise in the Social Sciences (FORS). https://www.swissubase.ch/en/catalogue/studies/13362/16860/overview. Accessed 27 Dec 2023

[CR99] Stolz J, Bünker A, Liedhegener A, Baumann-Neuhaus E, Becci I, Dandarova Robert Z, Senn J, Tanner P, Wäckerlig O, Winter-Pfändler U (2022). Religionstrends in der Schweiz: religion, spiritualität und säkularität im gesellschaftlichen Wandel.

[CR100] Stolz J, Huber F (2014) Wie kann man die Integration religiöser Gemeinschaften in die Gesellschaft erklären? In: Arens E, Baumann M, Liedhegener A, Müller WW, Ries M (eds.) Integration durch religion? Geschichtliche befunde, gesellschaftliche analysen, rechtliche perspektiven. Zürich, Pano, pp 21–40. https://www.academia.edu/en/6335238/Wie_kann_man_die_Integration_religi%C3%B6ser_Gemeinschaften_in_die_Gesellschaft_erkl%C3%A4ren_in_Arens_Edmund_Hgg_Integration_durch_Religion_Geschichtliche_Befunde_gesellschaftliche_Analysen_rechtliche_Perspektiven_RWP_Bd_10_Baden_Baden_Z%C3%BCrich_2014_S_21_40. Accessed 27 Dec 2023

[CR101] Stolz J, Könemann J, Purdie MS, Englberger T, Krüggeler M (2014) Google-Books-ID: qwU6CgAAQBAJ. In: Religion und Spiritualität in der Ich-Gesellschaft: Vier Gestalten des (Un-)Glaubens. Theologischer Verlag Zürich

[CR102] Szasz A (2015) We're pessimistic because we pay too much attention to climate deniers. In: AGU fall meeting abstracts, vol 2015, p ED13A-0884

[CR103] Taylor B, Van Wieren G, Zaleha B (2016). The greening of religion hypothesis (part two): assessing the data from Lynn White, Jr, to Pope Francis. J Study Religion Nat Cult.

[CR104] Taylor B, Wright J, LeVasseur T (2020). Dark green humility: religious, psychological, and affective attributes of proenvironmental behaviors. J Environ Stud Sci.

[CR105] Torabi M, Noori SM (2019). Religious leaders and the environmental crisis: using knowledge and social influence to counteract climate change. Ecum Rev.

[CR106] Tucker ME, Gottlieb Roger S (2006). Religion and ecology: survey of the field. The Oxford handbook of religion and ecology.

[CR107] Tucker ME (2008). World religions, the earth charter, and sustainability. Worldviews.

[CR108] Tucker ME, Grim JA (2001). Introduction: the emerging alliance of world religions and ecology. Daedalus.

[CR109] Veldman RG (2019). The gospel of climate skepticism: why evangelical Christians oppose action on climate change.

[CR110] Veldman RG, Wald DM, Mills SB, Peterson DAM (2021). Who are American evangelical Protestants and why do they matter for US climate policy?. Wires Clim Change.

[CR111] Wardekker JA, Petersen AC, van der Sluijs JP (2009). Ethics and public perception of climate change: exploring the Christian voices in the US public debate. Glob Environ Chang.

[CR112] White L (1967). The historical roots of our ecologic crisis. Science.

[CR113] Wilkins D (2020). Pope Francis, care for creation, and Catholic environmental imagery. Environ Hist.

[CR114] Willms C (2021) A cultural analysis of eco-Islam: How young German Muslims live religion through environmental activism. HTS Teologiese Studies / Theological Studies 77(2):11

[CR115] Wolkomir M, Futreal M, Woodrum E, Hoban T (1997). Denominational subcultures of environmentalism. Rev Relig Res.

[CR116] Woodrum E, Hoban T (1994). Theology and religiosity effects on environmentalism. Rev Relig Res.

[CR117] Woodrum E, Wolkomir MJ (1997). Religious effects on environmentalism. Sociol Spectr.

[CR118] World Council of Churches (1975). Breaking barriers. Nairobi 1975. The official Report of the Fifth Assembly of the World Council of Churches.

[CR119] Yin RK (2009) Case study research: Design and methods, vol 5. Sage. https://www.jstor.org/stable/23279888

[CR120] Zaleha BD, Szasz A (2015). Why conservative Christians don’t believe in climate change. Bull Atom Sci.

[CR121] Żuk P, Żuk P, Pluciński P (2021). ‘Coal basin in Upper Silesia and energy transition in Poland in the context of pandemic: the socio-political diversity of preferences in energy and environmental policy. Resour Policy.

